# Role of Hypoxia-Induced Brain Derived Neurotrophic Factor in Human Pulmonary Artery Smooth Muscle

**DOI:** 10.1371/journal.pone.0129489

**Published:** 2015-07-20

**Authors:** William Hartman, Martin Helan, Dan Smelter, Venkatachalem Sathish, Michael Thompson, Christina M. Pabelick, Bruce Johnson, Y. S. Prakash

**Affiliations:** 1 Department of Anesthesiology, Mayo Clinic, Rochester, Minnesota, 55905, United States of America; 2 Department of Physiology and Biomedical Engineering, Mayo Clinic, Rochester, Minnesota, 55905, United States of America; 3 International Clinical Research Center, Department of Cardiovascular Diseases, St. Anne's University Hospital, Brno, Czech Republic; 4 Department of Anesthesiology and Intensive Care, St. Anne's University Hospital, Masaryk University, Brno, Czech Republic; 5 Department of Internal Medicine, Division of Cardiovascular Medicine, Mayo Clinic, Rochester, Minnesota, 55905, United States of America; Niigata University, JAPAN

## Abstract

**Background:**

Hypoxia effects on pulmonary artery structure and function are key to diseases such as pulmonary hypertension. Recent studies suggest that growth factors called neurotrophins, particularly brain-derived neurotrophic factor (BDNF), can influence lung structure and function, and their role in the pulmonary artery warrants further investigation. In this study, we examined the effect of hypoxia on BDNF in humans, and the influence of hypoxia-enhanced BDNF expression and signaling in human pulmonary artery smooth muscle cells (PASMCs).

**Methods and Results:**

48h of 1% hypoxia enhanced BDNF and TrkB expression, as well as release of BDNF. In arteries of patients with pulmonary hypertension, BDNF expression and release was higher at baseline. In isolated PASMCs, hypoxia-induced BDNF increased intracellular Ca^2+^ responses to serotonin: an effect altered by HIF1α inhibition or by neutralization of extracellular BDNF via chimeric TrkB-Fc. Enhanced BDNF/TrkB signaling increased PASMC survival and proliferation, and decreased apoptosis following hypoxia.

**Conclusions:**

Enhanced expression and signaling of the BDNF-TrkB system in PASMCs is a potential mechanism by which hypoxia can promote changes in pulmonary artery structure and function. Accordingly, the BDNF-TrkB system could be a key player in the pathogenesis of hypoxia-induced pulmonary vascular diseases, and thus a potential target for therapy.

## Introduction

Diseases such as pulmonary hypertension (PH) are characterized by enhanced pulmonary artery (PA) constriction as well as arterial wall remodeling, the latter involving increased PA smooth muscle cell (PASMC) proliferation and survival but decreased apoptosis.[1,2,3] Accordingly, understanding mechanisms that alter PA remodeling and contractility is important.[1,4,5] Chronic hypoxia is a well-recognized contributor to PH[6,7] enhancing PASMC intracellular Ca^2+^ ([Ca^2+^]_i_) regulation and contractility[8,9] as well as cellular proliferation.[10,11] Therefore, the mechanisms by which hypoxia affects the PA become particularly relevant.

There is substantial interest in locally-produced growth factors (e.g. vascular endothelial growth factor) released within the PA in response to hypoxia with autocrine/paracrine effects on PASMCs.[12,13] In this regard, the family of neurotrophins, well-known in the nervous system,[14,15] may be relevant to the PA. Neurotrophins act via both high-affinity tropomyosin related kinase (Trk) and low-affinity p75NTR receptors, activating pathways including phospholipase C, PI3 kinase, mitogen activated protein kinases (MAPK), and NFκB.[15,16] They can genomically and non-genomically alter [Ca^2+^]_i_[17] as well as cell proliferation, survival and migration.[15,18] There is now increasing evidence (including our own) that neurotrophins such as brain-derived neurotrophic factor (BDNF) and their receptors (TrkB) are expressed and functional within the airways and pulmonary vasculature.[19,20,21,22] For example, Kwapiszewska et al. reported that BDNF/TrkB interactions can augment PASMC cell proliferation in idiopathic pulmonary hypertension. [23] In pulmonary endothelium and in airways, BDNF enhances [Ca^2+^]_i_ and NO generation[22] and in airway smooth muscle, BDNF promotes [Ca^2+^]_i_ responses[24] and cellular proliferation.[25] Further, hypoxia enhances PA endothelial cell (PAEC) synthesis and secretion of BDNF.[26,27]Given this, there is merit in further investigating the functional consequences of BDNF/TrkB expression in the PA, particularly following hypoxia exposure, and to determine how BDNF may interact with hypoxia signaling mechanisms to alter the structure and function of the PA.

In the brain, hypoxia increases BDNF expression,[28,29] and BDNF may modulate neuronal cell survival.[30] Similarly, BDNF has been shown to play a role in cardiac myocyte survival following infarction.[31] Furthermore, in neuroblastoma cell lines[32] as well as rat airway cell lines,[33] TrkB expression is mediated, in part, by exposure to low oxygen tensions. In this regard, the promoter of human NTRK2 gene, which encodes TrkB, contains at least three hypoxia-inducible factor (HIF) response elements.[34] Based on this information, we hypothesized that hypoxia enhances the BDNF-TrkB system in PASMCs, allowing BDNF to mediate and modulate the effects of hypoxia on arterial contractility and remodeling. The relevance of our study lies in demonstrating the potential importance of how BDNF/TrkB interactions could contribute to hypoxia effects in the PA, setting the stage for exploration of hypoxia-exacerbated diseases such as PH.

## Materials and Methods

### 1. Human PA and PASMCs

Under a Mayo Institutional Board-approved protocol, PASMCs were isolated from 3–4 mm diameter branch PAs of lung samples incidental to patient thoracic surgery at Mayo Clinic-Rochester. Samples were from lobectomies or pneumonectomies for focal non-infectious indications such as localized tumors, and were classified as being non-PH or PH based on clinical data. The limited PH data set was entirely mild-moderate, where mean PAP clinically estimated by transthoracic or transesophageal echocardiography ranged from 25 mmHg (mild) to 48 mmHg (moderate), given high anesthesia risk for non-transplant surgeries in severe PH. Samples were limited to areas distant from the focal pathologies as identified by the pathologist and verified under gross microscopy. Since the samples and clinical data were de-identified, the protocol was considered minimal risk Human Subjects Research.

Samples were transported rapidly in ice-cold Hank’s Balanced Salt Solution (HBSS). The PA segment was partitioned for testing of endothelium-intact PA, and for analysis of endothelium- and adventitia-denuded PA smooth muscle. The latter was used as tissue or for isolation of PASMCs, which involved mincing and growth of explants in DMEM with 10% FBS/1% ABAM. Tissue explants were maintained for 5–7 days at 37°C in 95% air/5% CO_2_ after which the source tissue was removed and cells plated in 60mm dishes (Western analysis), 8 well Labtek culture chambers or 96 well plates (imaging and proliferation assays). Prior to experimentation, cells were serum-deprived for 24h, and all cells were used between passages 1 and 4 to ensure maintenance of PASMC phenotype, verified by markers such as smooth muscle actin and myosin, and transgelin (SM22), but absence of fibroblast specific protein.

### 2. PA and PASMC Exposures

For hypoxia, intact PA or isolated PASMCs were exposed to 1% O_2_ for 48h in a hypoxia chamber (Biospherix C-chamber). The chamber was flushed 3 times for 10 min each with high flow 1% O_2_ prior to use. For BDNF exposure, intact PA or isolated PASMCs were incubated for 24 or 48 h (see protocols) at 37°C in regular growth media (vehicle control) or 100 pM recombinant human BDNF (#248BD, R&D Systems, Minneapolis, MN). This concentration was based on previous reports of circulating serum BDNF levels of ~20 ng/ml which translates to <1 nM. [35] [36]; [37] To determine the role of TrkB, PASMCs were incubated in 1μg/ml TrkB-Fc (Fc portion of TrkB receptor acting as extracellular BDNF neutralizing antibody, R & D Systems), ensuring adequate neutralization of nM concentrations of extracellular BDNF. The role of HIF1α was tested using an amidophenolic compound which blocks accumulation of HIF1α protein (preventing transcriptional activation) (#400083, EMD Millipore, 1 μM).

### 3. Immunofluorescence

Normoxia and hypoxia exposed human PASMCs were grown in serum free medium on 8 well slides with removable chambers. Medium was aspirated and cells were fixed in 2% paraformaldehyde for 15 min at room temperature, then washed 3 times in Tris buffered saline (TBS). Cells were then blocked with 5% donkey serum diluted in TBS for 1 h at room temperature on a shaker. Blocking solution was aspirated and primary antibody diluted 1:200 in TBS was added to appropriate wells. Control wells received no primary antibody. Primary antibody was incubated at 40°C overnight. Cells were then washed 3 times with TBS, and secondary antibody diluted 1:200 in TBS was added for 3 h at room temperature. Slides were finally washed, air dried, and coverslip mounted with DAPI Gelmount. Images were obtained using appropriate fluorescent wavelengths.

### 4. Serum/Extracellular BDNF Levels

Extracellular media from PA or PASMCs (see results) were processed for sandwich ELISA for BDNF (R&D Systems, Minneapolis, MN). Standard, manufacturer-provided protocols were used and colorimetric changes determined using a FlexStation3 microplate reader (Molecular Devices, Sunnyvale, CA) set to 450 nm (wavelength correction set to 540 nm) and compared to a standard, manufacturer-provided calibration curve.

### 5. Western Analysis

Standard SDS-PAGE (Criterion Gel System; Bio-Rad, Hercules, CA; 4–15% gradient gels) and PVDF membrane (Bio-Rad) transfer techniques were used. Membranes were blocked in buffer containing 5% powdered milk in TBS with 0.1% Tween-20 prior to addition of primary antibody (1 μg/mL rabbit anti-TrkB, Abcam, ab33655 or ab18987; rabbit anti-HIF1α, Novus Biologicals, #3376; rabbit anti-transgelin, Santa Cruz Biotechnology; sc-50446; mouse anti-β-actin, Abcam, AC-15). Markers of PASMC phenotype (smooth muscle actin, transgelin) were verified. As these PASMC markers, as well as β-actin, were relatively equally expressed in normoxic and hypoxic cells, we felt confident using β-actin expression as a measurable loading control. Protein detection and densitometry were done on Odyssey infrared imaging system (Li-Cor Biosciences) and all quantified bands were compared to the normoxia control band.

### *6*. Real Time Calcium Imaging

We have previously published techniques for imaging of cellular [Ca^2+^]_i_.[38] Briefly, cells were incubated in 5 μM fluo-4-AM (Invitrogen) at room temperature for 45 min and fluorescence measurements made on a FlexStation3 microplate reader with micropipetting of 1 μM serotonin. Fluorescence was empirically calibrated *in situ* by exposing PASMCs to known Ca^2+^ levels with the ionophore A-23187.

### 7. Cell Proliferation Assay

Proliferation of PASMCs was assayed at 48h (with or without preceding interventions) using the fluorescent CyQuant NF kit (Invitrogen) as previously described.[19] Cells were exposed to CyQuant dye for 1h at room temperature, and measurements taken on the FlexStation3. Dye calibrations were performed empirically using different cell counts in order to obtain estimates of baseline proliferation and with interventions over 48h. Extent of proliferation was normalized to baseline (i.e. time zero).

### 8. Statistical Analysis

PA tissue or PASMC experiments were performed in samples from 3–7 patients (no pooling), with 3 repeats/sample. Data were analyzed and results were compared using one way ANOVA with Student-Newman-Keuls or Dunnet’s *post hoc* analysis using SigmaPlot (SYSTAT, San Jose, CA), and statistical significance was established at *P* < 0.05. Values are means ± SE.

## Results

### 1. Hypoxia enhances PASMC-derived BDNF

**Fig 1:**
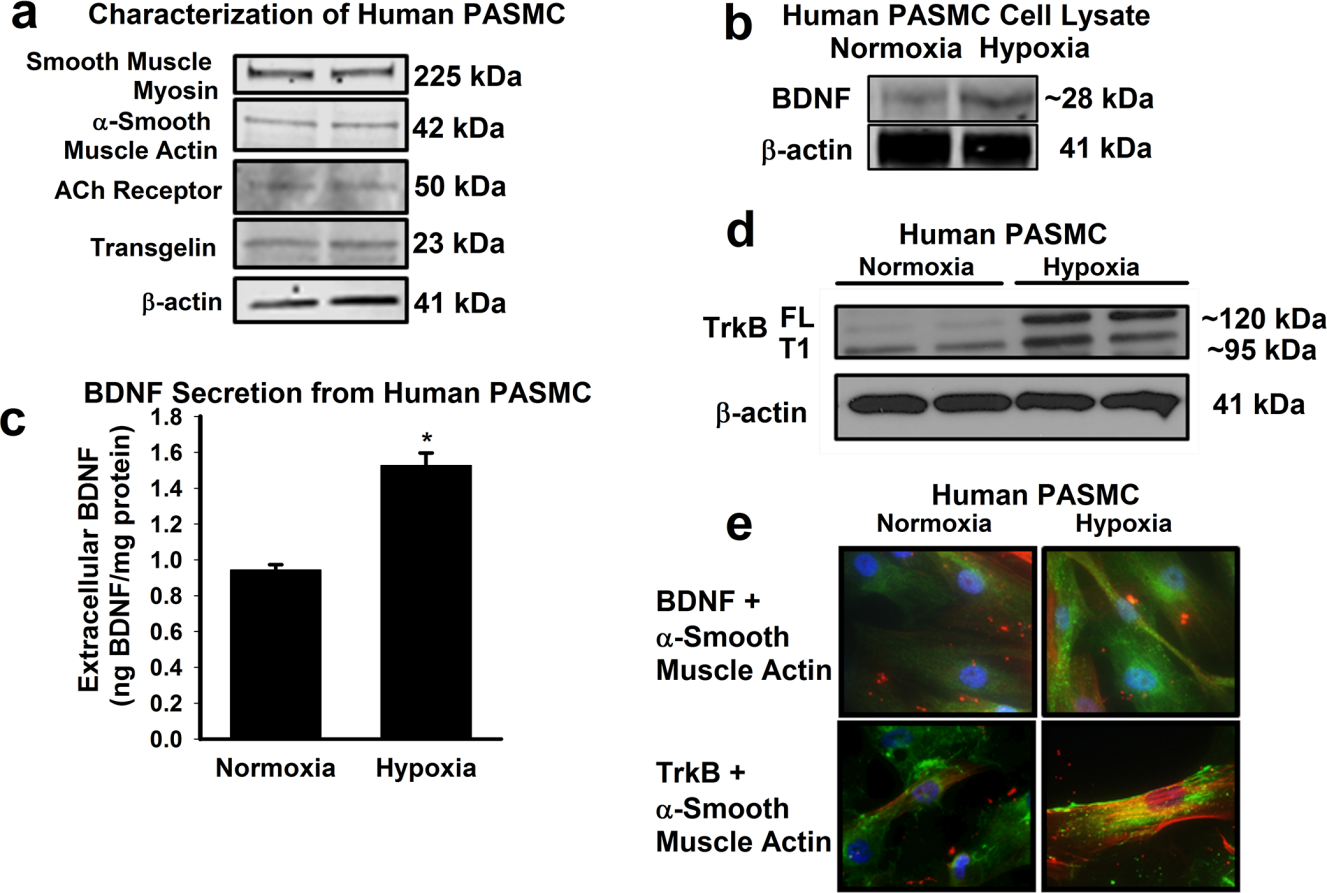
Characterization of human pulmonary artery smooth muscle cells (PASMCs). (a) Representative Western blot of proteins confirming PASMC phenotype including smooth muscle myosin, smooth muscle actin, acetylcholine receptor, and transgelin. Hypoxia enhances PASMC brain derived neurotrophic factor (BDNF) secretion. (b) Western blot analysis of human pulmonary artery smooth muscle cells (PASMCs) demonstrated presence of BDNF, with increased expression following 48h of 1% hypoxia. (c) Human PASMCs secrete BDNF, and such secretion is increased with hypoxia, as demonstrated by ELISA of supernatant in PASMC cultures. (d) Representative Western analysis showed increased TrkB expression (particularly full-length; FL) in PASMCs following hypoxia. (e) Immunocytochemistry demonstrating enhanced BDNF and TrkB expression in hypoxia-exposed human PASMCs compared to normoxia (green color represents BDNF or TrkB, red color represents α-smooth muscle actin, blue color (DAPI) represents nucleus staining. Values are means ± SE (n = 5 patients without pulmonary vascular disease). *Significant difference from normoxia (p<0.05).

Human PASMC were characterized by Western Blots demonstrating the presence of smooth muscle myosin, alpha smooth muscle actin, the acetylcholine receptor, and transgelin (Fig 1A). Western blot analysis of whole cell extracts of human PASMCs demonstrated the presence of BDNF with increased expression following hypoxia (Fig 1B, n = 3 patients without pulmonary vascular disease). In addition, immunohistochemistry demonstrated enhanced BDNF in hypoxia exposed PASMC compared to normoxia cells (Fig 1E). Importantly, ELISA of the supernatant from PASMCs exposed to normoxia vs. hypoxia (1% O_2_ which is known to induce HIF1α; see below)[8,39,40] demonstrated baseline presence of BDNF with increased secretion following hypoxia (Fig 1D; n = 7 patients without pulmonary vascular disease; p<0.05). In parallel Western blot studies, we found significantly increased full-length PASMC TrkB protein expression following hypoxia (Fig 1E; p<0.05). This increase in TrkB expression following hypoxia exposure further confirmed by immunofluorescence (Fig 1E).

### 2. Hypoxia enhances TrkB expression

**Fig 2:**
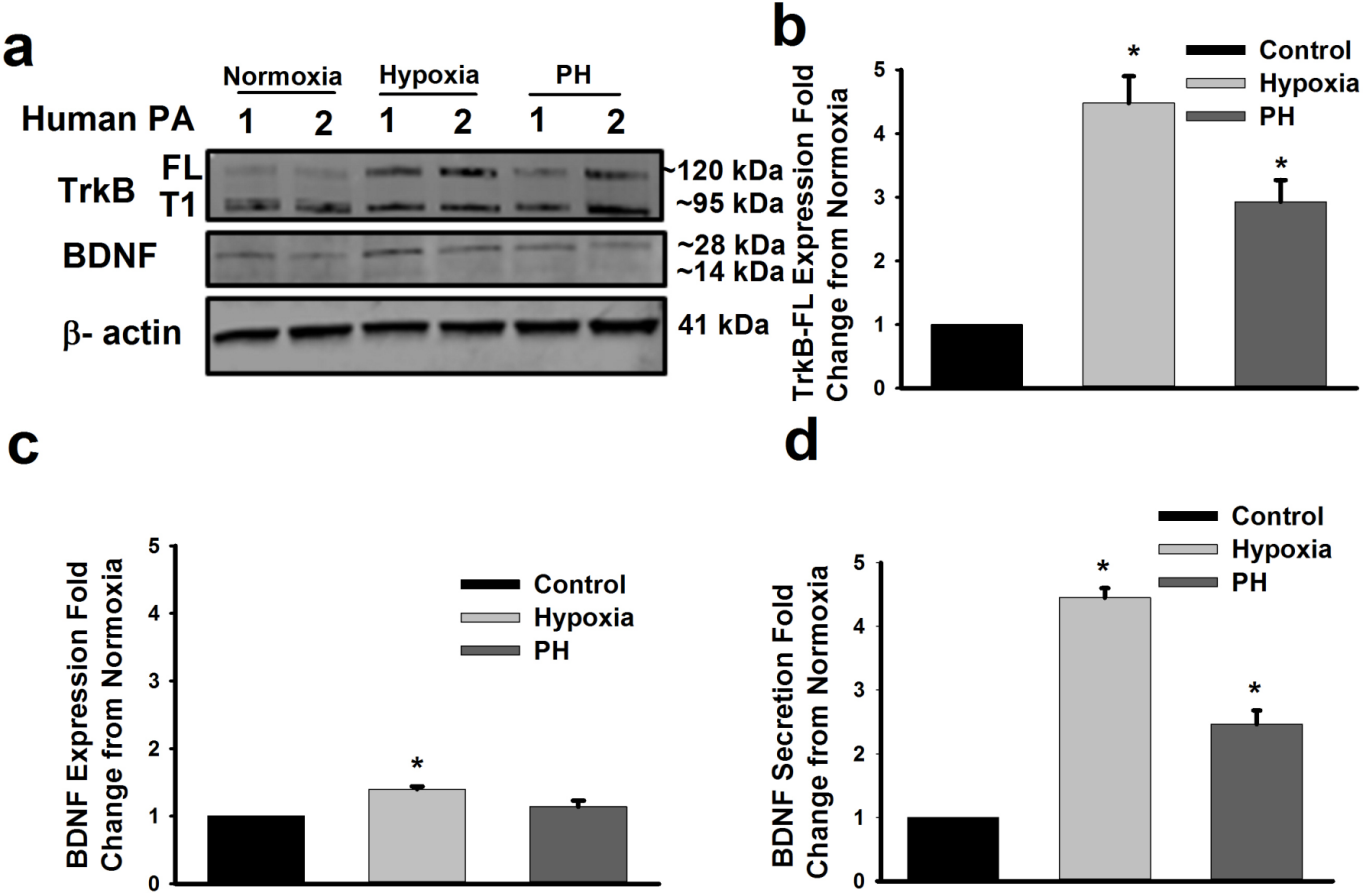
BDNF and its high affinity tropomyosin related kinase (TrkB) receptor are expressed in human pulmonary artery (PA). (a) Representative Western blots of endothelium-intact human PA show presence of both full length (FL) and truncated (T1) isoforms of TrkB. Exposure to 1% hypoxia increased TrkB-FL expression (a, b). Expression of TrkB-FL was also higher in PA of patients with moderate PH (a, b). BDNF expression in endothelium-intact human PA was also increased by hypoxia (c) albeit not to the extent as for TrkB. Importantly, ELISA of supernatants showed that human PA secretes BDNF and such secretion is enhanced by hypoxia and in PH (d). *Significant difference from normoxia (p<0.05). Values are means ± SE (n = 5 unique patients for normoxia and hypoxia and 3 unique PH patients in (b) and (c)).

Western blot analysis of endothelium-intact, normoxia vs. hypoxia-exposed PA segments (n = 5) from patients without pulmonary vascular disease, vs. normoxia-exposed PA segments from patients with moderate PH (Mean PAP clinically estimated by transthoracic or transesophageal echocardiography ranged from 25 mmHg (mild) to 48 mmHg (moderate)) (n = 3) showed that hypoxia as well as moderate PH resulted in increased TrkB expression, especially the full-length isoform (Fig 2A and 2B; representative blots of 2 samples from each group in (a), with average changes relative to normoxia in (b); p<0.05). Similarly, enhanced BDNF expression (pro- and mature isoforms) was observed in PA samples exposed to hypoxia, and those with PH, although these changes in BDNF were not as pronounced as with TrkB (Fig 2C). On the other hand, BDNF ELISA of supernatant media from these tissue samples demonstrated significantly enhanced BDNF secretion following hypoxia (compared to normoxia controls), as well as enhanced baseline BDNF secretion in PA tissues from moderate PH patients albeit to a lesser extent compared to hypoxia effects (Fig 2D; p<0.05).

**Fig 3:**
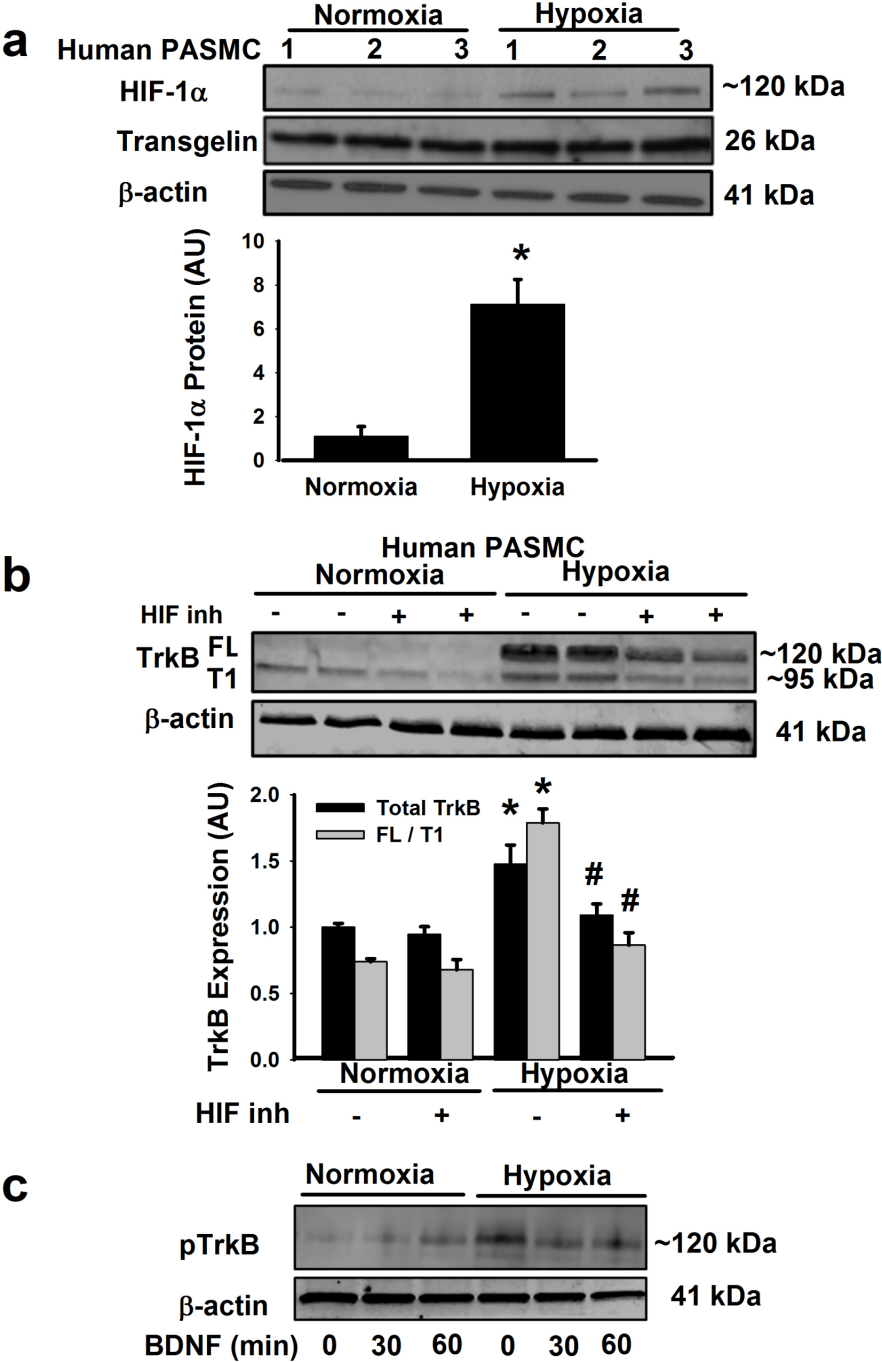
TrkB expression in PASMCs is enhanced by hypoxia. (a) In human PASMCs, 48h of 1% hypoxia enhanced expression of hypoxia-inducible factor (HIF-1α). Expression of β-actin or transgelin was not substantially altered showing maintenance of smooth muscle phenotype. (b) Hypoxia enhancement of TrkB was suppressed by pharmacological inhibition of HIF1α. Values are means ± SE (n = 7 patients). *Significant difference from normoxia, # significant effect of HIF-1α inhibition (p<0.05). (c) Western blot demonstrating phosphorylated TrkB expression in normoxia or hypoxia with exposure to BDNF for indicated times.

Western blot analysis of PASMC lysates from PAs of patients without pulmonary vascular disease showed increased HIF1α protein expression following 48h of 1% hypoxia (Fig 3A; p<0.05). Importantly, in the presence of a HIF1α inhibitor, hypoxia-induced increase in TrkB protein expression was suppressed (Fig 3B; p<0.05). Consistent with TrkB activity relying on phosphorylation, Western analyses showed enhanced pTrkB following addition of BDNF at 30 and 60 min exposure times in normoxia, albeit not to the same extent as when PASMCs were exposed to hypoxia. Subsequent addition of exogenous BDNF at 30 and 60 min did not enhance pTrkB levels compared to hypoxia alone (Fig 3C).

### 3. BDNF/TrkB in hypoxia effects on [Ca^2+^] responses of PASMCs

**Fig 4:**
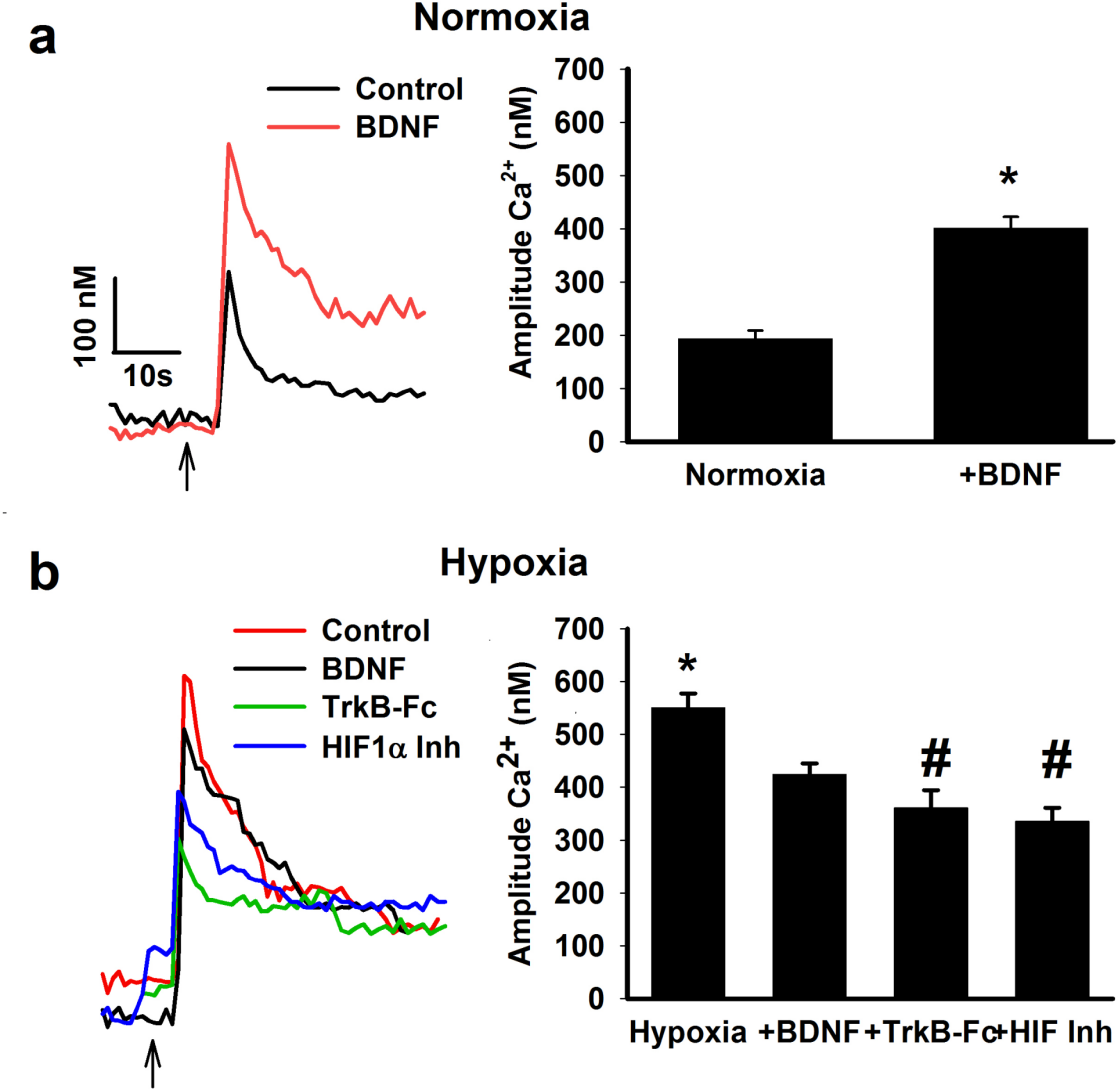
BDNF in hypoxia enhancement of intracellular Ca^2+^ ([Ca^2+^]_i_) responses to agonist in human PASMCs. (a) Exposure of PASMCs to 1 μM serotonin (5HT; arrow) resulted in a transient [Ca^**2+**^]_**i**_ response. Under normoxic conditions, 24h exposure to 100 pM BDNF enhanced the subsequent response to 1 μM 5HT, as reflected by increased amplitude (bar graph). (b) Hypoxia substantially enhanced [Ca^**2+**^]_**i**_ responses of PASMCs to 5HT. Under conditions of hypoxia, BDNF did not produce any additional augmentation of these responses. However, neutralization of extracellular BDNF using the chimeric TrkB-Fc (1 μg/ml) protein blunted the effect of hypoxia. Similarly inhibition of HIF1α reduced the effect of hypoxia on the responses to 5HT. Values are means ± SE (n = 7 patients). *Significant difference from normoxia control, #significant effect of inhibitors from hypoxia control (p<0.05).

Influo-4/AM loaded PASMCs of patients without pulmonary vascular disease, we found that 100 pM BDNF (in normoxia) enhanced the amplitude of [Ca^2+^]_i_ responses to 1 μM serotonin (5HT; Fig 4A; n = 5; p<0.05). In separate experiments, 5% hypoxia increased [Ca^2+^]_i_ responses to 5HT (Fig 4B; n = 5; p<0.05). Further addition of BDNF did not enhance responses to hypoxia. However, in the presence of the BDNF neutralizer, TrkB-Fc, hypoxia enhancement of [Ca^2+^]_i_ responses to 5HT was significantly blunted (p<0.05) but not eliminated. Furthermore, [Ca^2+^]_i_ responses to 5HT were diminished in the presence of the HIF1α inhibitor (Fig 4B; n = 5; p<0.05).

### 4. BDNF/TrkB in hypoxia effects on survival pathways

**Fig 5:**
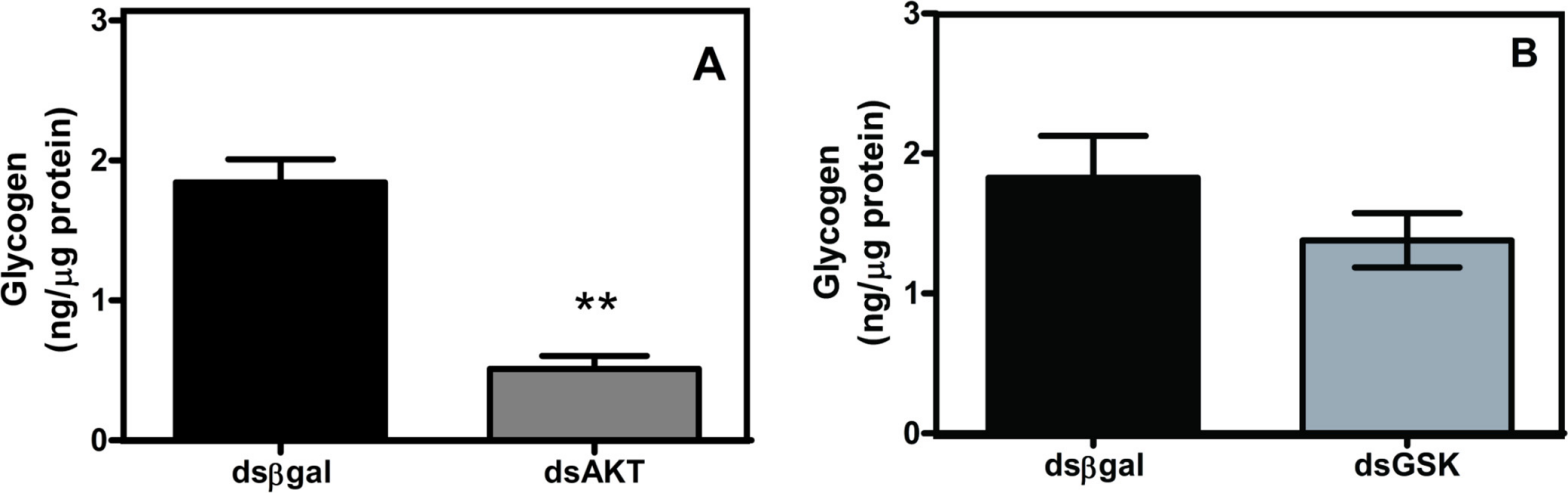
BDNF/TrkB in hypoxia effects on PASMC survival. Western blots for pPI3k, pAkt, and pERK in denuded human PA (aand b). After 24 hr hypoxia exposure, PI3 kinase (c) and pAkt/Akt (d) levels were both increased: effects blunted by BDNF neutralization or HIF1α inhibition. In normoxia, 100 pM BDNF enhanced PI3 kinase expression and Akt phosphorylation. 24h hypoxia also enhanced phosphorylation of ERK1/2 (e). In normoxia, 100 pM BDNF enhanced ERK1/2 phosphorylation. Extracellular neutralization of BDNF using TrkB-Fc (1 μg/ml) or inhibition of HIF1α blunted hypoxia effects on ERK phosphorylation. Values are means ± SE (n = 3 patients). *Significant difference from normoxia control; # significant inhibitor effect from hypoxia control (p<0.05).

Hypoxia has been previously shown to enhance PASMC survival and proliferation,[10,11,41] acting through pathways such as pPI3K and pAkt/Akt [25,42,43] which happen to overlap with BDNF signaling.[25] Representative Western blots are shown in Fig 5A and 5B. Following BDNF exposure in either normoxia or hypoxia, enhanced pPI3K and pERK until approximately 30 minutes of BDNF treatment were noted. No short term enhancement of pAkt was observed with BDNF. At 24h, however, (Fig 5B) enhanced phospho-protein expression was observed in the presence of BDNF, particularly pPI3K (Fig 5B) and pAkt (Fig 5C). Hypoxia alone also upregulated expression of these proteins. While the additional presence of BDNF did not further enhance pPI3K or pAkt/Akt levels in hypoxia, both the HIF1α inhibitor, and TrkB-Fc reduced expression of these pro-survival proteins in hypoxia exposed tissues (Fig 5B and 5C; n = 3 for each group; p<0.05 for BDNF, HIF1α inhibitor and TrkB-Fc).

In other cell systems, BDNF/TrkB signaling involves phosphorylation of ERK.[44,45] Western analysis of denuded PA tissue from patients (n = 3) without pulmonary vascular disease showed increased p-ERK/ERK protein levels in response to 24h BDNF exposure, comparable to that induced by hypoxia. Importantly, p-ERK/ERK levels were partially attenuated by the HIF1α inhibitor or by TrkB-Fc (Fig 5D; n = 3 for each group; p<0.05 for BDNF, HIF1α inhibitor and TrkB-Fc).

### 5. Role of BDNF in hypoxia effects on PASMC proliferation

**Fig 6:**
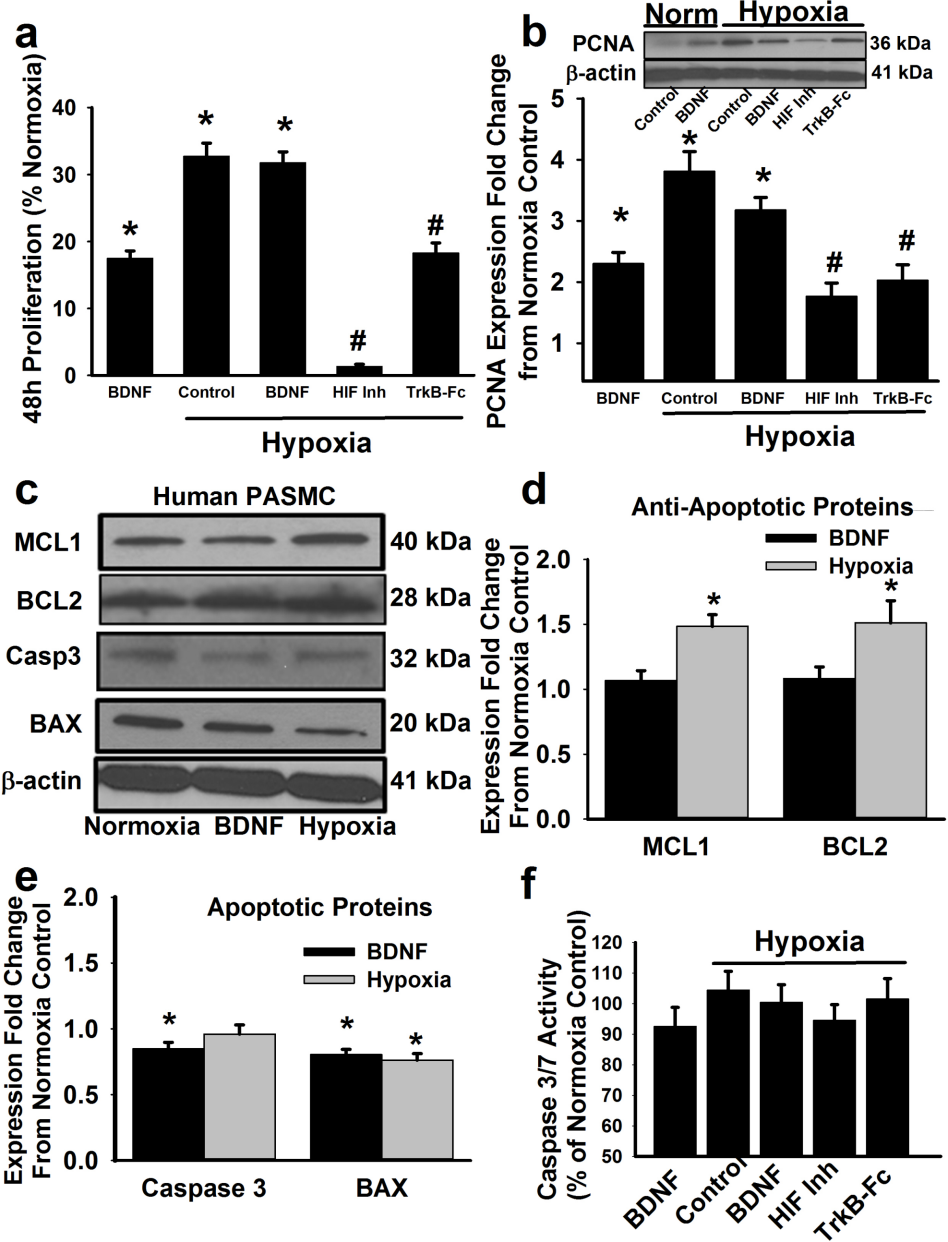
BDNF/TrkB in hypoxia effects on PASMC proliferation and apoptosis. PASMC proliferation was measured using a fluorescent CyQuant assay (a). Compared to baseline proliferation over 48h in normoxia (~6%), proliferation in BDNF exposed cells were substantially higher. Hypoxia by itself substantially increased proliferation, with no additional effect of BDNF. In contrast BDNF neutralization or HIF1 inhibition substantially blunted proliferation. The fluorescent proliferation assay results were matched by changes in proliferating cell nuclear antigen (PCNA) Western analysis (b). In contrast to the increase in proliferation, BDNF had only minimal effects on anti-apoptotic proteins MCL1 and BCL2 (c, d) but did reduce levels of the apoptotic proteins caspase 3 and BAX. Hypoxia effects were overall more pronounced for anti-apoptotic proteins, but less for apoptotic proteins. These protein changes were consistent with small changes in apoptotic activity as measured for caspase 3 vs. 7 (f). Values are means ± SE (n = 7 unique patients). *Significant BDNF (black band) or hypoxia (gray band) effect, #significant inhibitor effect (p<0.05).

An empirical calibration curve based on initially cell seeding concentrations and CyQuant fluorescence responsiveness [25] allowed comparisons of cell proliferation between groups. Serum-deprived PASMCs from patients without pulmonary vascular disease showed baseline proliferation of ~6% over a 48h period compared to time zero. Compared to baseline, PASMCs exposed to BDNF showed significantly greater proliferation (Fig 6A; p<0.05; n = 7 patients). Hypoxia alone also enhanced PASMC proliferation, and to a greater extent compared to BDNF only (p<0.05). The HIF1α inhibitor blunted hypoxia effects on proliferation. Furthermore, pre-treatment of hypoxia-exposed PASMC with TrkB-Fc also partially blunted PASMC proliferation (Fig 6A; p<0.05).

Proliferation was further verified by Western blot analysis of PASMCs for proliferating cell nuclear antigen (PCNA) following exposure to BDNF or hypoxia, both of which showed increased PCNA (Fig 6B; p<0.05 compared to controls). The HIF1α inhibitor as well as TrkB-Fc in the presence of hypoxia each reduced PCNA levels.

### 6. BDNF does not appear to diminish apoptosis marker expression

Increased cell numbers can represent enhanced proliferation and/or reduced apoptosis. Fig 6C and 6D show that expression of anti-apoptosis markers MCL-1 and BCL-2 was not significantly enhanced by BDNF. However, there was a significant decrease in the expression of the apoptosis proteins caspase 3 and BAX (Fig 6C and 6E; p<0.05). Hypoxia significantly enhanced anti-apoptosis protein markers, but decreased BAX (and not caspase 3). Consistently, a caspase 3/7 assay as a measure of apoptosis of PASMC exposed to normoxia vs. hypoxia (Fig 6F) showed no significant changes with BDNF or hypoxia.

## Discussion

Responsiveness of PASMCs to hypoxia plays an important role in PA structure and function, making the effects of such insults on PA relevant to pathogenesis of diseases such as pulmonary hypertension. In the present study, we demonstrated a potential contribution of a the neurotrophin BDNF, which is not only released by PAECs [26]and PASMCs in response to hypoxia but can also have autocrine effects of enhancing [Ca^2+^]_i_ responses and cellular proliferation that occurs with hypoxia. As reported here and by others, BDNF can promote PASMC proliferation.[23] The relevance of BDNF to hypoxia is suggested by increased circulating levels in human serum exposed to hypoxia, as well as enhanced PAEC expression and secretion of BDNF as reported previously by us [26]. In addition, others have demonstrated enhanced BDNF mRNA expression in hypoxia-exposed lung and heart cells.[27]Furthermore, higher expression of BDNF in PAs of patients with PH suggests a potential role for this neurotrophin in this disease. Accordingly, our results contribute to previous data [23] that neurotrophins may have autocrine/paracrine effects that can mediate and modulate hypoxia effects on the PA.

Neurotrophins are a family of small growth factors that signal via high affinity Trk receptors.[14,15] However, there is increasing evidence that neurotrophins, particularly BDNF, as well as Trk and low affinity p75NTR receptors are widely distributed in non-neuronal, peripheral tissues including lung. [46] Immunocytochemical and other evidence suggest that BDNF and TrkB are expressed by the pulmonary vasculature.[23,46,47,48] In this regard, the intima and adventitia have been shown to have higher expression compared to smooth muscle.[22,47] In previous studies, we found that BDNF is expressed by human pulmonary endothelial cells [49] [26]and that PAEC BDNF expression and secretion is enhanced in hypoxia. [26]Furthermore, we had reported that both the ligand and the full-length TrkB receptor are expressed, suggesting a functional BDNF/TrkB system. The results of the present study now show that in addition to PA endothelial cells, human PASMCs also express BDNF and more importantly TrkB receptor, highlighting another, important local source as well as target of BDNF in the PA. More importantly, such expression is increased by hypoxia. Here, the higher expression of BDNF in the PAs of patients with moderate PH may be relevant given the importance of hypoxia in PH, although much work is still necessary to establish the functional relevance of elevated BDNF in PH, or the relationship between hypoxia, BDNF and PH pathogenesis/progression. Nonetheless, data from serum samples in healthy humans exposed to hypoxia is particularly relevant. [26] Previous studies have reported circulating neurotrophins, particularly BDNF (e.g.[50,51]), with increasing evidence for altered BDNF levels in response to environmental and physiological stimuli.[52,53] In this regard, the serum BDNF levels observed in our study are comparable to other reports of ~20 ng/ml (which translates to <1 nM [35]; [54]; [37] justifying the use of a 100 pM concentration in our *in vitro* work).

While the present study, and our previous work [26] [22] highlight the presence of BDNF and its receptors in the PA, the functional relevance of BDNF/TrkB signaling warrants further investigation. We previously showed that BDNF works through TrkB to acutely (i.e. non-genomically) enhance endothelial NO production.[22] In other vascular beds, BDNF can increase eNOS expression and modulate endothelial cell proliferation, migration and survival via MAPK and PI3/Akt cascades.[55,56] These endothelium-based data would suggest that BDNF may facilitate vasodilation. However, it is currently unknown whether such a function is maintained in the setting of hypoxia or other factors such as inflammation. Our in vitro data showing that BDNF actually enhances [Ca^2+^]_i_ in PASMCs in the presence of hypoxia would suggest an opposing, vasoconstrictive effect that may offset any beneficial effects of BDNF on endothelium. Furthermore, the enhancing effect of BDNF on smooth muscle proliferation demonstrated in these data and reported by others [23] would only contribute to a thickened PA and increased contractility. In terms of [Ca^2+^]_i_ regulation, BDNF can activate mechanisms such as IP_3_ and influx pathways such as TRPCs, both of which are well-known to be important in PASMCs [8,57,58,59] and would contribute to increased contractility.

There are likely a number of mechanisms to explain how BDNF increases [Ca^2+^]_i_ responses to serotonin, including enhancement of Ca^2+^ influx as well as enhanced SR Ca^2+^ release. Furthermore, the enhancing effect of BDNF on agonist responses is probably not specific only to serotonin. For example, we have previously demonstrated in human airway smooth cells that BDNF can increase baseline [Ca^2+^]_i_ and responses to agonists such as Ach. [60]Further, with prolonged exposure, BDNF increases expression of a range of [Ca^2+^]_i_ regulatory proteins such as CD38, Orai1, IP_3_ and RyR receptors. [38]While BDNF did not affect baseline Ca^2+^ in PASMCs, it is possible that prolonged BDNF exposure could indirectly increase [Ca^2+^]_i_ by altering expression of regulatory proteins: a topic yet to be examined.

Separately, BDNF can promote an anti-apoptotic phenotype by enhancing MCL-1[61] and BCL-2.[62] Hypoxia can induce anti-apoptotic proteins in PASMCs.[63] We found that BDNF tended to enhance expression of MCL-1 and BCL-2, and reduce caspase expression. These data support the idea that BDNF/TrkB signaling in human PASMC can contribute to hypoxia-induced effects in multiple ways.

Overall, these data point to a complex role of locally-produced BDNF within the PA that may detrimentally or beneficially influence arterial contractility and relaxation in a context-sensitive fashion. A single study in pulmonary artery rings from p75NTR knockout mice showed increased contractions to endothelin-1[64] suggesting a vasodilatory role for neurotrophins, however such an effect cannot be attributed to BDNF (p75NTR is activated by all neurotrophins), to TrkB, or to PASMCs. Furthermore, it is likely that long-term lack of p75NTR could have a completely different effect on vascular structure and function.

Our studies show that hypoxia increases PASMC BDNF secretion resulting in autocrine effects, as demonstrated by neutralizing effects of the chimeric TrkB-Fc. Furthermore, hypoxia increases TrkB receptor expression, facilitating autocrine BDNF effects, particularly in the setting of enhanced BDNF release. The mechanisms by which hypoxia enhances BDNF or TrkB expression are under investigation. While HIF1α would be an obvious choice, there are no HIF1α responsive elements on the BDNF gene. Whether hypoxia enhances BDNF secretion by other, indirect mechanisms including NFκB or MAPK is not known and warrants further investigation. On the other hand, there are at least three possible HIF1α response elements on the TrkB gene promoter, NTRK2.[34] In accordance, pharmacological inhibition of HIF1α reduced TrkB protein expression in PASMCs.

In terms of signaling, binding of BDNF results in TrkB autophosphorylation and triggering of a number of downstream enzymes and intracellular signaling cascades such as MAP kinases and PI3/Akt.[15,16] It is likely that the range of signaling mechanisms is both cell and context dependent. In human airway smooth muscle, we previously showed that MAPK, PI3/Akt and NFκB are all activated by BDNF, particularly with inflammation.[19] The present results showing activation of PI3/Akt and ERK are entirely consistent in this regard.

In conclusion, our study demonstrates enhancement of BDNF and TrkB expression and signaling in PASMCs following hypoxia, resulting in increased PASMC [Ca^2+^]_i_ and proliferation that can potentially contribute to hypoxia effects on arterial contractility and remodeling. While locally produced BDNF may be particularly important, the potential exists for circulating BDNF to further influence PA. Future studies should examine the mechanisms by which hypoxia can modulate endothelial vs. PASMC BDNF/TrkB signaling and the overall, *in vivo* effect on PA contractility and remodeling, especially in the context of diseases such as PH.
